# Oral bacteriophages and their potential as adjunctive treatments for periodontitis: a narrative review

**DOI:** 10.1080/20002297.2025.2469890

**Published:** 2025-02-25

**Authors:** Mwila Kabwe, Joseph Tucci, Ivan Darby, Stuart Dashper

**Affiliations:** a Department of Rural Clinical Sciences, La Trobe Rural Health School & La Trobe Institute for Molecular Science, La Trobe University, Bendigo, Victoria, Australia; b Melbourne Dental School, University of Melbourne, Parkville, Victoria, Australia

**Keywords:** *Fusobacterium nucleatum*, dysbiosis, *Treponema denticola*, *Porphyromonas gingivalis*, phage therapy

## Abstract

**Background:**

There is no specific cure for periodontitis and treatment is symptomatic, primarily by physical removal of the subgingival plaque biofilm. Current non-surgical periodontal therapy becomes less effective as the periodontal pocket depth increases and as such new adjunctive treatments are required. The development of antibiotic resistance has driven a recent resurgence of interest in bacteriophage therapy.

**Methods:**

Here we review the published literature with a focus on the subgingival phageome, key oral pathobionts and the dysbiotic nature of periodontitis leading to the emergence of synergistic, proteolytic and inflammophilic bacterial species in subgingival plaque. We discuss the opportunities available, the barriers and the steps needed to develop bacteriophage therapy as an adjunctive treatment for periodontitis.

**Results:**

The oral phageome (or virome) is diverse, featuring abundant bacteriophage, that could target key subgingival bacteria. Yet to date few bacteriophages have been isolated and characterised from oral bacterial species, although many more have been predicted by genomic analyses. Bacteriophage therapy has yet to be tested against chronic diseases that are caused by dysbiosis of the endogenous microbial communities.

**Conclusion:**

To be effective as an adjunctive treatment for periodontitis, bacteriophage therapy must cause the collapse of the dysbiotic bacterial community, thereby resolving inflammation and enabling the reestablishment of a health-associated mutualistic subgingival bacterial community. The isolation and characterisation of novel oral bacteriophage is an essential first step in this process.

## KEY MESSAGES

1. The oral phageome is still poorly described and characterised especially with regard to lytic bacteriophage.
2. Bacteriophage therapy has yet to be applied to any chronic human disease caused by microbial dysbiosis.
3. Despite many challenges bacteriophage therapy offers the potential to be a safe, specific and efficacious adjunctive treatment for periodontitis.

## Periodontitis

Periodontitis, formerly described as chronic periodontitis, is a bacterial induced inflammatory disease of the supporting tissues of the tooth that is characterised by the progressive destruction of those tissues and is regarded as the most common chronic inflammatory, non-communicable human disease. The Global Burden of Disease 2010 study reported the global age-standardized prevalence (1990–2010) of severe periodontitis to be 10.5–12.0%, representing the sixth-most prevalent condition in the world [1]. Moderate-to-severe forms of periodontitis affect approximately 30% of adults [2], whilst the prevalence of all forms including milder disease may be as high as 50% [2,3]. Periodontitis is characterised by loss of the supporting structures of the teeth, and is diagnosed using clinical attachment loss (CAL) and alveolar bone loss, the presence of periodontal pocketing and gingival bleeding [4,5]. Diagnosis is made after destruction.

Although periodontitis is largely preventable and controllable in the majority of cases, if untreated, it can lead to tooth loss and impaired quality of life [6]. Periodontitis is a chronic disease that progresses over years, during which time, the diseased supra- and sub-gingival epithelia are exposed to heavy bacterial loads. With increasing disease severity, periodontitis can become a significant source of systemic as well as localised inflammation and a source of persistent bacteraemia. The disease has been linked to an increased risk of diabetes, cardiovascular diseases, certain cancers, pre-term birth, rheumatoid arthritis and Alzheimer’s disease [7,8].

In 2018, the direct costs of periodontitis were estimated to be US$3.5 billion in the US and €2.5 billion in Europe. Indirect costs resulting from periodontitis were US$150 billion in the US and €156 billion in European countries [9]. These data indicate that periodontitis is a major public health problem globally with a substantial economic and social burden.

## Current treatments

There is no specific cure for periodontitis to address the underlying microbial cause and treatment is symptomatic. The recent Clinical Practice Guidelines, developed under the auspices of the European Federation of Periodontology, recommend that initial clinical treatment of periodontitis should be subgingival instrumentation (also known as non-surgical mechanical debridement or scaling and root planning). This is aimed at reducing the subgingival plaque biofilm and calculus, leading to a reduction in probing pocket depths, gingival inflammation and the number of diseased sites [5]. This therapy is recommended for all periodontitis patients, irrespective of their disease severity, for those teeth with loss of periodontal tissue support and/or periodontal pocket formation. In patients who are motivated to maintain a high level of oral hygiene, this treatment can result in long-term periodontal stability [10,11]. However, depending upon the evaluation criteria used, up to 40% of diseased sites may not respond well to treatment [12,13]. Factors that contribute to a failure of treatment include residual calculus and anatomical features of the site that limit accessibility to the tooth root preventing thorough plaque removal [14–16]. In general, subgenival instrumentation becomes less effective as the periodontal pocket depth increases [17–19]. In these sites, Retamal-Valdes *et al.* [20] concluded that subgingival instrumentation alone was unable to produce a sufficiently significant change in the oral microbial ecology in order to produce a new stable climax biofilm community compatible with periodontal health. Therefore, a range of adjunctive therapies has been proposed with the purpose of potentiating the effects of subgingival instrumentation.

Sanz *et al.* [5] evaluated the adjunctive interventions to subgingival instrumentation, including: physical or chemical agents, host-modulating agents (local or systemic), subgingival locally delivered antimicrobials, or systemic antimicrobials. Of 12 specific adjunctive treatments the panel considered, the only ones they cautiously recommended were some of those that modulated the bacterial components of the subgingival plaque. The panel consensus was to not recommend the use of nine adjunctive treatments, namely photodynamic therapy or lasers, local administration of statin gels (atorvastatin, simvastatin, rosuvastatin), probiotics, systemic sub-antimicrobial dose doxycycline, locally delivered bisphosphonate gels or systemic bisphosphonates, systemic or local non-steroidal anti-inflammatory drugs, omega−3 polyunsaturated fatty acids, and local administration of metformin gel; due to lack of evidence regarding efficacy or a cost to benefit analysis. The use of adjunctive antiseptics, specifically chlorhexidine mouth rinses for a limited period of time, and locally administered sustained-release antibiotics was recommended for consideration. However, these have a number of side effects that may prevent long-term use, such as staining. Although the combination of metronidazole and amoxicillin has been consistently shown to offer significant clinical and microbiological benefits as an adjunctive treatment [21] due to concerns about the patient’s broader health and the impact of systemic antibiotic use to public health, its use as a routine adjunct to subgingival debridement in patients with periodontitis was not recommended [5].

Management of periodontal disease is more than just initial removal of bacterial deposits, but occurs over the long term, i.e many years. Maintenance of improvements in periodontal health and prevention of breakdown are managed by regular recall appointments and supportive periodontal therapy. These appointments involve removing the biofilm that has accumulated between visits and motivating patients to continue or improve their oral hygiene. Even within a maintenance program there can be continued progression and attendance at these appointments decreases over.

Given the failure of up to 40% of sites to respond well to subgingival instrumentation, the chronic nature of the disease and the current lack of specific and acceptable adjunctive treatments there is clearly a need for development of novel adjunctive treatments that address the root cause of periodontitis, subgingival plaque bacterial community structure and composition. The manipulation of periodontal microbiota has the potential to serve as an adjunct or an alternative to conventional mechanical periodontal therapy [22]. The goal of this adjunctive treatment would be to tip the balance in favour of the re-establishment of eubiotic health-associated communities [22,23]. This adjunctive treatment would need to be specific and not target bacterial species associated with health. A recent small clinical trial of the probiotic *Lactobacillus reuteri* has shown some potential as an adjunctive treatment in periodontitis possibly due to its ability to inhibit pathogenic community reformation and its anti-inflammatory properties [24]. Bacteriophage therapy (below) offers the potential for high specificity targeting of key pathobionts with no known side effects.

## Microbiology of periodontitis

**Figure 1.**
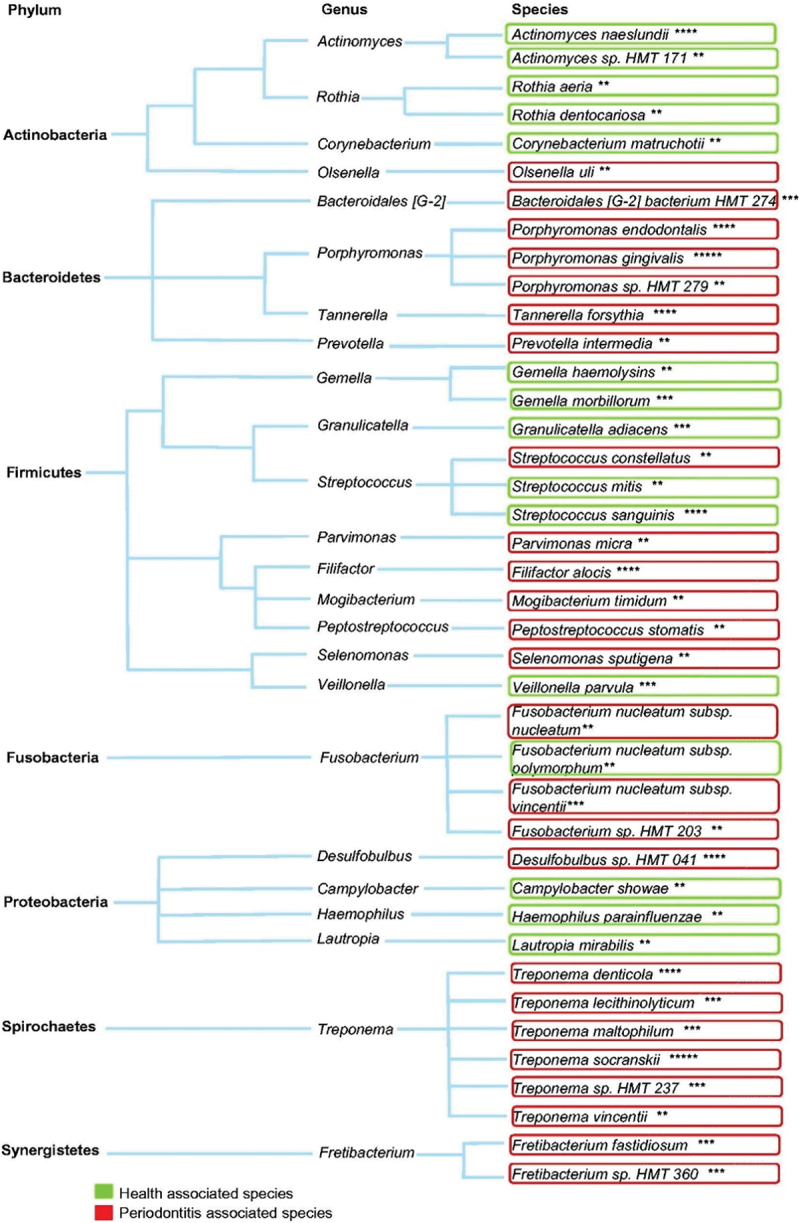
The major health and periodontitis-associated oral bacterial species found in subgingival plaque. The data was derived from analysis of seven individual studies and the number of asterisks refers to the number of studies that identified the health or disease association of each species. Reprinted from Balan et al. [22].

The microbial aetiology of periodontitis is complex. Periodontitis is a site-specific inflammatory disease that is now widely accepted to be caused by a microbial dysbiosis in subgingival plaque biofilms. This results in the emergence of a limited number of pathobionts, oral bacteria that exist in the oral cavity during health but under specific environmental conditions induce chronic disease [25]. These pathobionts, including *Porphyromonas gingivalis, Treponema denticola, Fusobacterium nucleatum*, *Filifacter alocis* and *Tannerella forsythia*, come to dominate the bacterial community of subgingival plaque at the base of the periodontal pocket (Figure 1). They increase substantially in the subgingival plaque biofilm prior to tissue breakdown and alone can exceed 25% of the subgingival plaque microbiota [13,23]. There is also a large increase in the number of bacteria in the subgingival sites due to the expansion of the site, and the increased flow of nutrients. In addition, the neutralisation of an effective host response also favours bacterial proliferation.

There is still considerable discussion in the scientific literature around the importance of specific bacterial species to the development of the dysbiotic bacterial community associated with periodontitis with, for example, *P. gingivalis* having been described at various times as an opportunistic pathogen [26], a key component, along with *Treponema denticola*, *Tannerella forsythia* of the disease-associated Red Complex [27], a keystone pathogen [28,29] and now as part of the pathogenic dysbiotic subgingival bacterial community [30].

Bacterial interactions within a dysbiotic community are proposed to be fundamental processes affecting chronic human disease initiation, progression, severity, susceptibility to antimicrobial therapy and resolution. These polymicrobial biofilm communities consist of multiple cells cooperating to build a differentiated structure, and as such exhibit the emergent characteristics of multicellular organisms. Within polymicrobial biofilms particular species have specialised functions and different metabolic rates associated with specific developmental profiles [31]. This is especially true of oral polymicrobial biofilms that can be very complex and long lived. Specialisation is needed for bacteria to build complex biofilms due to the relatively limited size of bacterial genomes. One of the defining features of complex polymicrobial biofilms is the production of an extracellular matrix that can act not only to protect biofilm cells from environmental stressors but can also provide a defined structure and help to spatially locate particular species within the biofilm. A small number of recent studies indicate that micron-scale spatial structures resulting from interspecies bacterial adhesion and metabolic interactions are key determinants of the function of polymicrobial biofilm communities [32,33]. The tools to further define these spatial structures are now being developed which will enable these observations to be further characterised [34]. With regard to periodontitis species such as *Fusobacterium nucleatum* and *Streptococcus gordonii* are thought to enable pathogenic plaque community development acting as scaffolding and symbiotic partners [35,36], although *F. nucleatum* may also play a direct role in disease progression. Sakanaka *et al.* [37] have recently shown *F. nucleatum* produces putrescine from arginine via ornithine that accelerates the biofilm life cycle of *P. gingivalis*. Similarly, *P. gingivalis* has been shown to release diffusible signals that enhance *F. nucleatum* biofilm development [38]. These types of cooperative metabolism within oral biofilms can tip the balance toward periodontitis [37]. Indeed, *F. nucleatum* may be seen as a keystone species in dysbiotic community development due to its ability to coaggregate widely with both early colonising health associated species and species associated with disease, and its flexible metabolic capabilities, although this remains to be tested [35,37]. More of the fundamental metabolic processes underpinning subgingival bacterial community development and stability are now being characterised at least between a limited number of species in these communities. *Streptococcus gordonii* for example has been proposed as important for the colonisation of a site and persistence of *P. gingivalis*, especially in health, due to its syntrophic ability, symbiotic biofilm formation and protection from oxidative stress [36]. Syntrophic interactions and avoidance of substrate competition between *P. gingivalis* and *T. denticola* have been shown to greatly increase biomass when the species are co-cultured [39]. The motility of *T. denticola* along with the metabolic interactions have been shown to play crucial roles in polymicrobial biofilm development with *P. gingivalis* [40–42].

**Figure 2.**
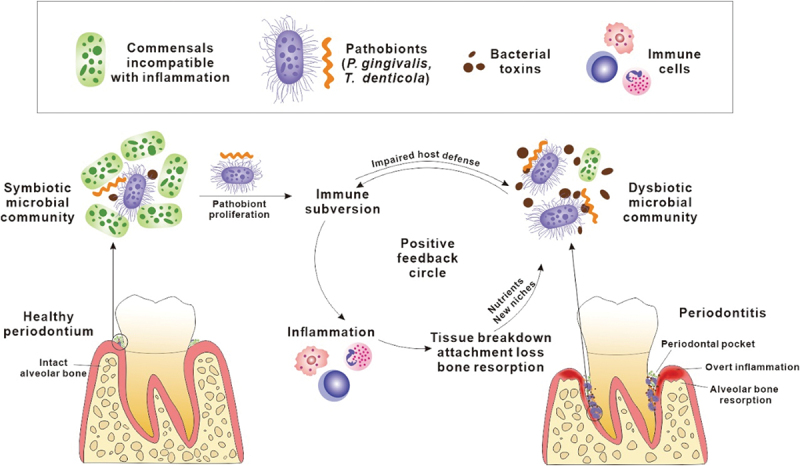
Development of a dysbiotic subgingival plaque community during periodontitis. The dysbiotic microbial community which is characterized by an increase in abundance of a small number of pathobionts stimulates local host inflammatory responses. Inflammation results in tissue breakdown that produces nutrients, including peptides, haem and ferrous iron, further facilitating the proliferation of inflammophilic pathobionts. The unresolved inflammation deepens periodontal pockets providing more suitable habitat for pathobionts. Reprinted with permission from Liu et al. [23,43].

**Figure 3.**
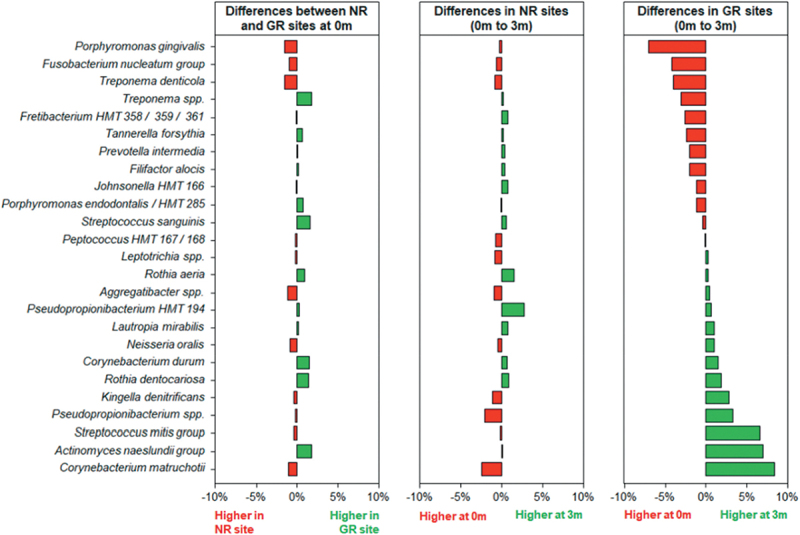
Differences in the relative abundances of the 25 most abundant bacterial taxa at a species level in subgingival plaque prior to and in response to non-surgical debridement treatment in sites with no response (NR) and matched good responding (GR) sites. Subgingival plaque samples were taken just prior to treatment (0 m) and three months after treatment (3 m). The x-axis depicts the relative change in abundance as a percentage of the total. Reprinted with permission from Byrne et al. [23].

This dysbiotic community works collaboratively and symbiotically to cause host inflammation, dysregulate that response and subvert it to provide a range of macro- and micro-nutrients (Figure 2). Although there is likely to be some redundancy in this community, the removal of a small number of species may have broader implications on the whole community. The ability to disrupt the micron-scale spatial structures of these disease-associated communities by targeting key species involved in community physical structure or in essential metabolic processes offers the potential to collapse these communities and may enable the re-establishment of health associated communities. This contention has not been widely tested due to the lack of specific agents that can selectively remove single species from dysbiotic communities and the use of specific agents, such as bacteriophages, could greatly improve our understanding of the dynamics of these bacterial communities. Oxantel pamoate, which has no effect on *T. denticola*, was shown to collapse polymicrobial biofilm development with *P. gingivalis* and *T. forsythia* inhibiting all three species to a similar degree, demonstrating the synergistic nature of biofilm development by these species and the dependence of *T. denticola* on the other two [44]. In actively progressing periodontal pockets *F. nucleatum* was the most abundant bacterial taxa at a species level, followed by *P. gingivalis* and *T. denticola*. All these species decreased significantly in abundance in those sites that responded to treatment but remained high in those sites that did not respond to treatment (Figure 3 [23]).

The establishment of pathogenic bacterial communities in periodontal pockets that are closely associated with an ulcerated epithelial layer enables these bacteria and their products to enter the bloodstream and circulate to distal parts of the body. *F. nucleatum* and *P. gingivalis* are now referred to as oncobacteria, that have been linked to the development, metastasis and chemotherapy resistance of oropharyngeal, pancreatic, colorectal and breast cancers [45,46]. *F. nucleatum* has also been associated with preterm birth [47]; *P. gingivalis* has recently been shown to be involved in the development of atherosclerosis, rheumatoid arthritis, Alzheimer’s disease [8,43,48,49] and *T. denticola* has been implicated in Alzheimer’s disease amongst other systemic diseases and disorders [50]. Consequently, limiting the proliferation of these bacterial species in the oral cavity may also have significant human health benefits outside the oral cavity.

## Bacteriophages

Bacteriophage (from the Greek ‘bacterium eater’) are viruses that are obligate intracellular parasites of bacteria. They are the most diverse and numerous biological semiautonomous entities on the planet, estimated to number in excess of 10^30^ individual virus particles [51]. Bacteriophages play as yet poorly defined roles in bacterial ecology, but some estimates describe a high turnover of bacteria by bacteriophages.

## Bacteriophage classification

While classification of bacteriophages by the International Committee on Taxonomy of Viruses (ICTV) is under revision due to expansion and diversity of characterised viruses [52], morphologically, bacteriophages exist as tailed, non-tailed and filamentous forms (Table 1). Filamentous bacteriophages belong to the class *Faserviricetes* and order *Tubulavirales*, which is composed of three families, *Inoviridae, Plectroviridae* and *Paulinoviridae* [ICTV (accessed 31/01/2023]. Tailed bacteriophages now classified as *Caudoviricetes* are commonly isolated and characterised, and exist in three main morphotypes, (i) Siphovirus: characterised by an icosahedral capsid and a long and flexible tail (ii) Myovirus: characterised by an icosahedral capsid and a rigid but contractile tail, and (iii) Podovirus consisting of an icosahedral capsid and short tail. Currently, a significant proportion of sequenced tailed bacteriophages remain unclassified at the family-level and a diverse group of non-tailed bacteriophages remain ungrouped into order or class [ICTV (accessed 31/01/2023)].

**Table 1.** Classification of bacteriophages.

Nucleic acid type	Class	Morphology and bacteriophage families	Illustration and electron micrograph
Tailed (dsDNA)	Caudoviricetes	***Myoviruses***: *Ackermannviridae, Aggregaviridae, Assiduviridae, Chaseviridae, Herelleviridae, Hafunaviridae, Kyanoviridae, Molycolviridae, Soleiviridae, Halomagnusviridae, Peduoviridae, Pyrstoviridae, Soleiviridae, Vertoviridae, and Winoviridae*	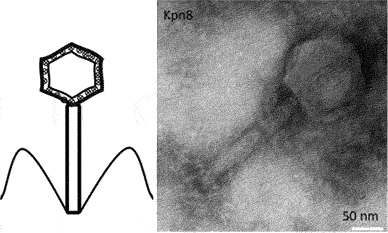 [53]
		***Siphovirus**: Anaerodiviridae, Casjensviridae, Demerecviridae, Drexlerviridae, Druskaviridae, Duneviridae, Haloferuviridae, Helgolandviridae, Forsetiviridae, Graaviviridae, Leisingerviridae, Madisaviridae, Mesyanzhinovviridae, Naomviridae, Orlajensenviridae, Vertoviridae, Suolaviridae, Saparoviridae, Straboviridae, Vilmaviridae, and Zierdtviridae*	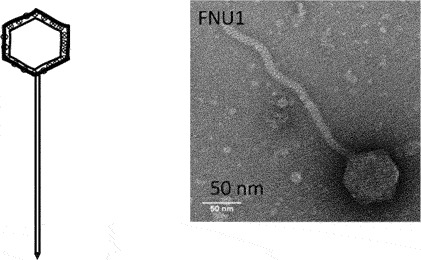 [54]
		***Podovirus**: Autographiviridae, Crevaviridae, Guelinviridae, Intestiviridae, Pachyviridae, Pervagoviridae, Rountreeviridae, Salasmaviridae, Schitoviridae, Steigviridae, Shortaselviridae, and Zobellviridae*	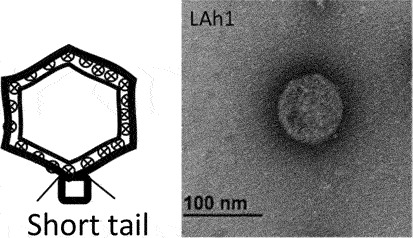 [55]
Non-tailed (dsDNA)	Tectiliviricetes	**Icosahedral virions, ~57 nm** *Corticoviridae*	Spike proteins 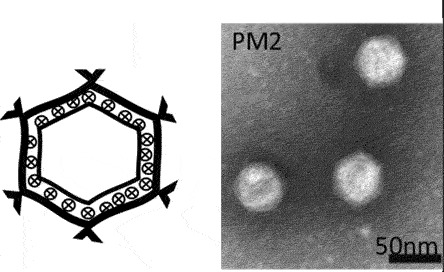 [56]
		**Non-envelope with flexible spikes, ~66 nm** *Tectiviridae*	Flexible spike proteins 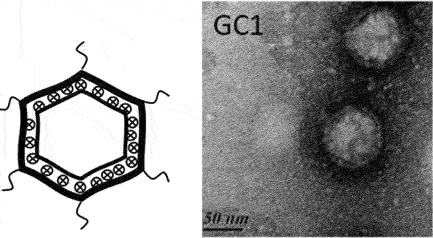 [57]
	*Not classified*	**Slightly pleomorphic, enveloped viruses, 50–125 nm***Plasmaviridae*	Lipid membrane 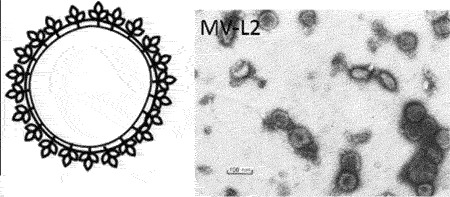 [58]
ssDNA	Malgrandaviricetes	**Microvirus** *Microviridae*	Mushroom shaped protrusions 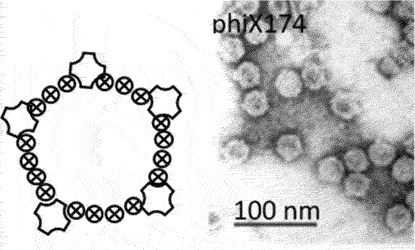 [59]
	Faserviricetes	**Flexible filamentous virus, ~ 7 nm diameter** *Inoviridae* *Paulinoviridae*	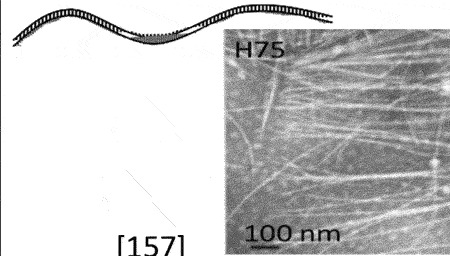 [59]
		**Non-enveloped rigid rods** *Plectroviridae*	
dsRNA	Vidavericetes	**Enveloped spherical virus, ~ 85 nm diameter** *Cystoviridae*	Surface protein 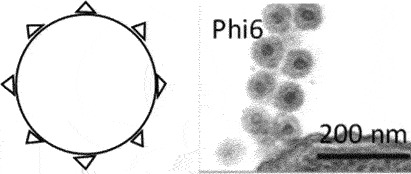 [59]
ssRNA	Leviviricetes	**Non-enveloped spherical virus, ~26 nm diameter** *Fiersviridae*	Maturation protein 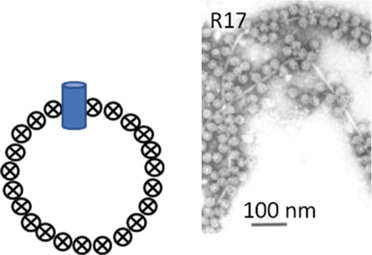 [59]

Bacteriophages exist as either double or single stranded DNA or RNA viruses. The majority of isolated and sequenced bacteriophage genomes contain double stranded (ds) DNA [60]. At approximately 4.4–500 kb [61] in size, their genomes are small, in comparison to other microbes (bacterial genomesfor example, are in the range of 160 kb for microbacteria to 10 Mb for macrobacteria [62,63]). This factor makes them very efficient replicating entities. The bacteriophage genome is composed of clusters of operons or cassettes, usually in the order of genes encoding proteins involved in lysogeny, DNA replication, regulation of transcription, DNA packaging and head assembly, tail, and lysis functions [64,65].

There are two main bacteriophage lifestyles. Lytic bacteriophages do not integrate into the bacterial DNA and cause cell lysis following replication, releasing more copies of themselves in the process. In the lysogenic life cycle, prophages or temperate bacteriophages integrate their DNA into host chromosomal DNA and their genomes are replicated along with that of the host [66]. In their lysogenic form, these bacteriophages may enhance the persistence and virulence characteristics such as biofilm formation of the host bacteria [67]. Lytic bacteriophages may produce endolysins that digest the cell wall when exiting the host bacteria [68]. Certain temperate bacteriophages have been shown to undergo the lytic cycle when induced in the laboratory or spontaneously. For instance, bacteria containing prophages may be exposed to ultra-violet light or cytotoxic agents such as mitomycin C and these environmental insults induce the temperate bacteriophage to undergo the lytic cycle when host bacteria are exposed to potential DNA damage. This DNA damage may be unrepairable triggering cell SOS responses [69].

## Bacteriophage therapy

Bacteriophage therapy involves the targeted application of bacteriophage(s) that, upon encounter with specific pathogenic bacteria, infect and kill them. Their initial application for treatment of bacterial infections was independently described by Frederick Twort in 1915 and two years later by Felix d’Herelle [70–72]. Although their therapeutic use continued in Eastern Europe (Russian and Georgia) [73], interest in Western countries waned due to inconsistent and variable efficacy observed earlier on and the discovery of penicillin by Alexander Fleming in 1929 [74]. However, the emergence of the antimicrobial resistance pandemic [75] has renewed the interest of therapeutic bacteriophages in many parts of the world including Australia, Europe, America and Africa where bacteriophage banks [76] and clinical trials are being established [77].

Bacteriophage(s) lyse the bacteria, releasing virion progeny that continue the cycle. Bacteriophages are unique among antibacterial agents in their ability to increase their numbers when in the presence of bacterial targets. Of similar importance, bacteriophages only minimally impact non-target bacteria or body tissues [78]. Displaying a narrow spectrum of activity can be a useful property for an antibacterial [79], and this feature makes bacteriophages an attractive option in instances where precision therapy is required. For a detailed history of the use of bacteriophage therapy to treat human infections beginning shortly after the discovery of bacteriophage and extending to recent times see Abedon *et al.* [78]. A recent meta-analysis of early clinical trials of bacteriophage therapy conducted in the first half of the 20^th^ century using a random effects model has demonstrated efficacy in the treatment of a wide variety of bacterial infections [80]. A recent review of bacteriophage clinical trials has indicated that while efficacy has not been reported in all clinical trials, all modern trials thus far have shown bacteriophage therapy to be safe [81].

## Bacteriophage isolation, choice and purification

Bacteriophages have co-evolved with bacteria and can be readily isolated from their bacterial host’s habitats [82]. Lytic bacteriophages may be filtered from natural samples and sequenced as a purified bacteriophage population or as phageome through metagenomics. Lytic bacteriophages are preferred for use as anti-bacterial agents. It is therefore important that therapeutic bacteriophages do not undergo a lysogenic lifecycle or possess genes encoding toxins, antibiotic resistance or others that may enhance the pathogenicity of the target or commensal bacteria [83]. However, as lytic bacteriophages may not always be isolated [84], the temperate bacteriophages in the genomes of bacteria [85] may provide an option for therapy. These prophages may possess lysin genes, and the subsequent proteins may be purified and utilised as antimicrobials [86]. In clinical applications, preparations of bacteriophages and/or lysins need to be rid of contaminants such as endotoxins derived from their host bacteria [87].

## Bacteriophages for treatment of biofilm-forming bacteria

The vast majority of bacteria exist as components of polymicrobial biofilms with complex interactions and ecologies, especially in the human oral cavity. High levels of bacteriophage release in biofilms have been demonstrated and the resulting lysis of cells may provide nutrients allowing the persistence and proliferation of neighbouring cells as well as remodelling the structure of the biofilm [88,89].

Bacteriophages carry specific polysaccharide depolymerases that can degrade capsular polysaccharides, exopolysaccharides or lipopolysaccharide aiding viral penetration of complex bacterial biofilms and bacterial capsules [90]. Bacteriophages have been shown to have the capacity to diffuse through mono- and multi-species biofilms [91], possibly through the penetration of the extracellular polymeric matrix known to house water and nutrient channels and poles [92]. Kabwe *et al.* [93] demonstrated the efficacy of bacteriophage application to *F. nucleatum* monospecies biofilms. Apart from *in-vitro* and *ex-vivo* treatments in a human root canal model targeting *Enterococcus faecalis* [94], bacteriophages have not been applied to treat oral disease. However, bacteriophages have successfully been used to treat other biofilm infections in humans, although exact compositions of bacteriophage preparations have not been completely reported. In wound infections, 13 studies reviewed by Duplesis and Biswas [95] (342 patients in total) reported efficacy ranging from 70% to 100%. Genevière *et al.* [96] reviewed 20 studies comprising 51 patients with a bacteriophage success rate of 71% in prosthetic joint and bone infections, while more recently, four patients have been successfully treated for chronic relapsing cardiovascular implant infections, one with a polymicrobial infection including MDR *Pseudomonas aeruginosa* [97]. The efficacy and safety of a bacteriophage cocktail treatment for antibiotic resistant *Pseudomonas aeruginosa*-associated chronic otitis was determined in a controlled double-blind Phase I/II clinical trial [98]. A phage cocktail containing six bacteriophages was applied directly into the ear of 12 patients whilst a control group of 12 patients received a placebo. A significant improvement in clinical indicators of disease, a decrease in *P. aeruginosa* counts after treatment, and no adverse events, were reported.

## Engineered bacteriophages and bacteriophage-based products

In addition to ‘native’ bacteriophage, engineered bacteriophage and their products offer opportunities for the development of novel therapeutic approaches. Well-characterised bacteriophages have been used for molecular biology applications due to their highly manipulable genomes [99]. However, bacteriophage engineering has also shown potential to assist in treatment of disease. For instance, prophage manipulation into lytic bacteriophage forms has been applied for treatment of disseminated drug-resistant *Mycobacterium abscessus* [100]. In other studies, bacteriophages have been engineered to modify their tail proteins, with which they recognise their host, to i) target emerging bacteriophage resistant mutants [101], ii) produce polyvalent bacteriophages that target different bacterial hosts [102,103] and iii) carry broad-spectrum antimicrobial peptides [104] for purposes of antimicrobial therapy or bacterial detection.

Upon attachment, bacteriophages may release enzymes known as ectolysins to facilitate the injection of their DNA into the bacteria [105]. During infection, these bacteriophages also express endolysins that target and lyse the bacterial cell wall peptidoglycan to release bacteriophages from inside the bacteria [106]. Although lytic bacteriophages are preferred as antimicrobials, they may not always be easily isolated [84]. Yet the genomes of many bacteria contain lysogenic prophage genomes [85]. These prophages may provide a library of proteins including lysins that could be repurposed as antimicrobials, potentially targeting the dysbiotic molecular pathways seen in chronic diseases [86]. Indeed, prophages have been described for three major periodontal pathobionts: *F. nucleatum* [107], *P. gingivalis* [108] and *T. denticola* [67].

Engineered bacteriophage can also be used as delivery vehicles for exogenous molecules using a process known as bacteriophage display. In this process peptides and/or proteins are expressed on the capsid of a bacteriophage, that is used to delivered the payload to a specific site [99]. This can be achieved through fusion of desired protein genes to the coat protein gene of the bacteriophage [109]. The displayed protein may be a peptide acting as an agonist or antagonist for receptor–ligand interaction or as vaccine carriers for known antigens [110]. Bacteriophage display has also been used to carry antibodies used for treatment of autoimmunity [111], a snake bite venom [112], viral infection [113] and allergic reactions [114].

## Human oral phageome

The contribution of bacteriophages to the composition and function of the human microbiome has been largely overlooked until recently. A detailed exploration of the human oral phageome is beyond the scope of this review and is covered in an excellent recent publication by Szafrański *et al.* [115]. Carr *et al.* [116] characterised the phageomes of 633 human oral sites using nucleic acid sequencing approaches. The host specificity of the bacteriophage can be inferred from the host genome sequence at the site where the virus integrates into the genome and by fragments of host DNA that are acquired by the bacteriophage and transferred between bacterial cells. The greatest bacteriophage diversity was found on the tongue and in plaque. Notably, 37 unique circular jumbo bacteriophage genomes were identified in oral sites, particularly on the dorsum of the tongue. Most oral metagenomes contained a high abundance of *Caudoviricetes*. Guo *et al.* [117] recently reviewed the roles of oral bacteriophages in a range of oral diseases and concluded that given the number of characterised bacteriophages specific for *E.s faecalis* there was significant potential for bacteriophage therapy development targetting endodontic infections associated with this bacterium. A recent systematic review encompassing 14 individual studies found that recent studies were highly heterogenous and tended to focus on the whole oral phageome. This led to difficulties in synthesizing and comparing the data, but overall indicated that there were associations between oral bacteriophages specific for *Streptococcus*, *Actinomyces*, *Haemophilus* and *Veillonella* and the oral diseases chronic periodontitis and caries [118].

## Known bacteriophages for periodontal pathobionts

A recent review of the human oral phageome [115] used the Integrated Microbial Genome/Virus (IMG/VR) database to predict bacteriophages specific for oral bacterial species. The 4th version of the IMG/VR database catalogues >15 million uncultivated viral genomes (UViGs) including both DNA and RNA viruses, identified as viral contigs or integrated proviruses in genomes, metagenomes, or metatranscriptomes, all systematically annotated and available through the IMG/VR user interface (https://img.jgi.doe.gov/vr/) [119]. Despite the prediction of these bacteriophage genomes in the oral phageome, reports of isolation of bacteriophages for oral pathobionts are rare. These bacteria have been shown to not thrive outside their natural habitat. This is in contrast to *Enterobacteriaceae* such as *Klebsiella* [120], and as such, bacteriophages against this group are readily isolated from external environments such as wastewater sources [82]. Further, oral pathobionts such as *Porphyromonas gingivalis* [121] and *Streptococcu*s *mutans* [122] have been shown to possess an extensive range of genes coding for CRISPR (clustered regularly interspaced short palindromic repeats) and CRISPR spacer sequences. It is also important to note that occasional differences between the genomes of predicted prophages and those that can be induced in the laboratory occur and may reflect the fact that some prophages may not be easily induced using current methods. For instance, some of the prophage genomic sequences previously identified in the genome of *Pseudomonas aeruginosa* were not detected after induction of prophages with norfloxacin or boiling bacterial suspensions [123]. This suggests that there is room for refinement of methods for characterising lysogeny, and improvement of both in-silico and experimental analyses, to better understand prophage diversity and activity [85].

Below are key bacteria taxa, mainly at a species level, that are associated with the progression of periodontitis based on the recent comprehensive review by Balan *et al.* [22] and the microbiomic analysis of nonresponding sites after mechanical debridement ([23]; Figure 3). Currently, available information regarding specific bacteriophages for these bacteria is presented.

### Bacteroidetes

Most currently predicted or isolated bacteriophages of the Bacteroidetes infect commensal species with only a small number known to infect species associated with disease progression [115].

*Porphyromonas gingivalis* - to date there are no reports of the isolation of bacteriophages lytic for this species, or closely related species, such as *Porphyromonas endodontalis*, that have also been strongly associated with disease progression. Matrishin et al. [124] using a bioinformatic *in silico* approach focused on temperate phage discovery found taxonomically diverse prophages are common in *P. gingivalis* being found in approximately a third of the examined genomes. This may explain the abundant prevalence of CRISPR-Cas systems in this species [121]. CRISPR-Cas systems are known to identify and target specific foreign DNA sequences, acting to limit bacteriophage infection. Fragments of bacteriophage DNA become incorporated into the bacterial genome on CRISPR memory arrays, and RNA probes transcribed from these arrays can identify the complementary invading bacteriophage DNA and guide antiphage bacterial nucleases.

*Tannerella forsythia* is predicted to host at least four distinct bacteriophages by the IMG/VR database [115], to date none of these have been isolated.

*Bacteroidales* [G-2] bacterium HMT 274 - no bacteriophages have been reported to be associated with this taxon although 23 are predicted to infect *Bacteroidales* [F-2] [G-2] HMT-272 [115]. The imprecise nature of the taxonomy of uncultivated oral bacterial species may confound the prediction of bacteriophage specificity based purely on genomic sequence data and it is possible that some of the 23 predicted bacteriophages of *Bacteroidales* [F-2] [G-2] HMT-272 May infect *Bacteroidales* [G-2] bacterium HMT 274.

*Prevotella intermedia* − 19 bacteriophages have been found to be associated with *P. intermedia/Prevotella nigrescens* based on sequence data [115] although there are no reports of active bacteriophages to date.

### Spirochaetes

The genus *Treponema* contains a relatively large number of species that are associated with periodontitis and none that are associated with health. A number of oral treponemes have been closely associated with disease, the best studied of which is *Treponema denticola*, followed by *Treponema socranskii. Treponema lecithinolyticum*, *Treponema maltophilum* and *Treponema* sp. HMT 237 (*medium*) [125].

To date a single bacteriophage Ψtd1 has been isolated and characterised from a single oral spirochaete, *T. denticola* [67]. The bacteriophage was initially discovered as a prophage in the bacterial genome and subsequently shown to be inducible and lytic. Similar to previously discovered spirochaete bacteriophages Ψtd1 is a tailed, double-stranded DNA, temperate bacteriophage that has been observed to have a contractile tail, indicating a Myovirus morphotype [67,126]. The IMG/VR database includes two oral bacteriophages that are predicted to infect *T. socranskii* [115].

### Fusobacteria

The species *Fusobacterium nucleatum* consists of several subspecies most of which have been associated with disease. The four best characterised subspecies are *F. nucleatum nucleatum, F. nucleatum animalis, F. nucleatum polymorphum* and *F. nucleatum vincentii*. None of these subspecies are differentiated in 16s RNA gene microbiomic studies, possibly limiting our understanding of their specific roles in disease and health.

**Figure 4.**
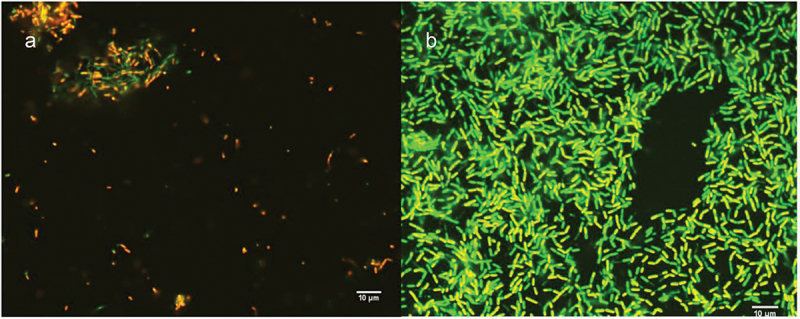
Confocal images of SYBR® gold and propidium iodide staining following FNU1 bacteriophage treated (a) and untreated (b) *Fusobacterium nucleatum* biofilm. Reprinted with permission from Kabwe et al. [60].

A total of 147 bacteriophages, including 26 jumbo bacteriophages, are linked to *Fusobacterium* spp. through sequence analysis although the species are not well identified [115]. Kabwe *et al.* [93] isolated and characterised FNU1 from *F. nucleatum polymorphum*. FNU1 is a large *Siphoviridae* virus with a genome of 130,914 bp, a capsid diameter of 88 nm and a tail of ~310 nm in length that was shown to disrupt *F. nucleatum* biofilms (Figure 4). FNU1 has been proposed as a potential treatment for *F. nucleatum*-mediated diseases and conditions including colorectal cancer [127]. More recently, Wang *et al.* [128] isolated five *F. nucleatum* myoviruses from saliva of healthy individuals (JD-Fnp1, JD-Fnp2, JD-Fnp3, JD-Fnp4) and faecal samples from a colorectal cancer patient (JD-Fnp5). Cochrane et al. [107] isolated two prophages from *F. nucleatum animalis* and designated them ɸFunu1 and ɸFunu2. ɸFunu2 was not able to bind to *F. nucleatum* and induce active lysis. Using bioinformatics approaches, they predicted the presence of up to 39 bacteriophages in the genomes of 16 *F. nucleatum* strains although the majority of these were incomplete or questionable, indicating possible limitations of prediction software.

### Firmicutes

*Filifactor alocis* has no known bacteriophage, although this may be due to minimal experimental efforts in discovery of these.

### Synergistes

The IMG/VR database includes the draft genome sequences of two oral bacteriophages, one of which is a jumbo bacteriophage, that are predicted to infect *Fretibacterium fastidiosum* [119].

### Proteobacteria

*Desulfobulbus* sp. HMT 041 has no known or predicted bacteriophage to date.

## Potential for bacteriophages as adjunctive treatment in periodontitis

A curated mixture of bacteriophages may have efficacy in the treatment of periodontal pockets that do not respond to conventional mechanical debridement. These could potentially be applied in a slow release form to all treated pockets at the time of treatment. Mechanical debridement will substantially disrupt the subgingival bacterial biofilms enabling a high level of penetration for the bacteriophage. Although lack of understanding of bacteriophage pharmacokinetics and pharmacodynamics has been described as a major hurdle to clinical translation [129], bacteriophages have shown favourable outcomes when used as adjunct to other existing therapies in a range of diseases. When used in conjunction with antibiotics, bacteriophage T4 has been shown to have enhanced burst size and to reduce the effective minimum biofilm eradication concentration of cefotaxime against *Escherichia coli* [130]. Further, bacteriophage/antibiotic synergy has been shown to extend susceptibility in previously antibiotic resistant pathogenic *E. coli* harbouring chloramphenicol acetyltransferase or beta-lactamase enzymes [131] and bacteriophage resistance [132]. Efficacy and synergy of bacteriophage and antibiotic therapy has also been shown in longer term clinical applications. After 3 years of continuous ceftazidime therapy, Chan *et al.* [133] were able to wean a 76-year-old patient with a *P. aeruginosa* mediastinal and aortic graft infection off the antibiotics by administering bacteriophage OMKO1 together with ceftazidime. Similarly, Ferry and colleagues, after treatment failure with prolonged systemic antibiotic therapy, used a cocktail of three bacteriophages in addition to surgery and antibiotic therapy to manage three elderly patients with *S. aureus* infected prosthetic knee infections [134]. Although highly complex and capable of avoiding the development of bacterial resistance mechanisms through evolutionary processes there still remains the possibility that overuse or incorrect use of bacteriophages will result in the resistance being developed [8]. Some bacteria such as *Listeria monocytogenes*, *E. faecalis* and *Acinetobacter baumannii* are known to convert to capsule-deficient forms in order to develop bacteriophage resistance [135,136]. This however, renders target bacteria susceptible to antibiotics [135] and less virulent [137]. Further, while bacteriophage and antibiotic synergy has been observed in many applications, some studies have shown minimal or antagonistic effects. For instance, using the bacteria *P. aeruginosa*, no synergy was observed between the bacteriophage KPP25 and any antibiotic tested [138]. Antagonistic effects with bacteriophages and antibiotics have also been observed in *E. coli*, *S. aureus*, *Bacillus cereus* treatments [139]. It is unclear why observed interactions between bacteriophages and antibiotics vary.

## Potential application, delivery and regulation of bacteriophage therapy in periodontal disease

As shown by Byrne et al. [23] the microbiome profile of the periodontal sites is an important prognostic marker. The population of non-responders or relapsing patients would be the target group for trialling the effectiveness of bacteriophages in periodontitis. In this case, a cocktail of bacteriophages specifically targeting identified bacterial pathobionts would be ideal. We have previously shown that bacteriophages can be formulated into a range of dosage forms, including (tooth) pastes and lozenges [140], however to be effective in the treatment of periodontitis a specific subgingival application is required. For subgingival application, bacteriophage suspensions could be directly applied during the debridement process, and subsequently. Dental floss impregnated with lyophilised bacteriophages could allow activation of the viruses in the moist subgingival environment [141]. This diverse range of therapeutic delivery forms could assist in limiting local inflammation and damage induced by the oral pathobionts, as well as their spread to distant sites where they may contribute to other serious chronic diseases.

## Potential risks for the development of bacteriophage therapy

A potential issue when considering bacteriophage therapy is the risk of host bacteria developing resistance and reduced susceptibility to lysis by the virus. Bacteria may mutate and evolve to overcome the threat from the lytic bacteriophage. They also possess sophisticated innate defence systems, including restriction-modification and CRISPR-Cas systems [142]. Therefore, bacterial defence systems, along with mechanisms for bacteriophage to counter them, play a role in bacteriophage resistance. Approaches for overcoming bacteriophage resistance include allowing bacteria and virus to co-evolve, the use of genetic engineering, co-treatment with antibiotics, and targeting biofilm-forming pathways such as quorum sensing [143].

Some bacteriophages have been shown to induce an inflammatory immune response when used in therapy, accompanied by bacteriophage-specific antibodies [144]. In the oral cavity, secretory immunoglobulin A (sIgA) is the primary immunoglobulin [145], and bacteriophage therapy has been shown to induce treatment limiting humoral immunity via sIgA in the gut [144]. As such, it may be prudent for bacteriophages used in periodontitis therapy to be tested against the patient’s sera before administration. In immune-compromised patients, bacteriophages have been shown to be safe [146].

The narrow host range/spectrum of bacteriophages provides both an advantage and a disadvantage. In acute critical care, this may be a disadvantage as there will be the requirement for screening of bacteriophages for their specificity. In such circumstances, broad spectrum antibiotics may be considered. However, in treatment of periodontitis, the precision and specificity of bacteriophages provides a valuable weapon for targeting pathobionts without affecting the healthy microbiota in the process.

Antibiotic resistance genes may be transferred between bacteria through horizontal gene transfer. It is possible that with extensive bacteriophage therapy, resistance to bacteriophages may be similarly transferred. In the past decade or so, many previously unknown prokaryotic defence systems have been characterised [142]. However, because bacteriophages are very specific to their hosts, it has been argued that it is unlikely that increased exposure will result in widespread bacteriophage resistance [147].

The success of bacteriophage therapy depends on its safety and efficacy. Bacteriophages are cultured on bacterial hosts and this process leads to production of bacterial debris known as endotoxins. As with other therapeutics, bacteriophages need to be tested for safety. In parenteral administration, less than 5 endotoxin units/kg body weight is permitted, and reproducibility is required to meet Good Manufacturing Practices [148]. Because of their delicate nature, crude bacteriophage manufacturing processes may lead to structural damage and loss of efficacy [149]. Bacteriophages for the treatment of periodontitis will need to meet regulatory hurdles associated with clinical trials prior to approval [150]. Currently, there are no approved regulations for bacteriophage treatment. However, with increasing evidence of their therapeutic capacity, many governing bodies such as Australian Therapeutic Goods Administration, European Medicines Agency and the United States Food and Drug Administration are becoming more amenable to approving the compassionate and magistral use of bacteriophages in therapy [151,152]. Regulation following a framework set out for similar therapeutic goods [153,154] e.g. attenuated virus vaccines, may facilitate the use of bacteriophages in patients.

## Conclusions

In the long-term bacteriophage therapy offers the potential to be a safe, specific and efficacious adjunctive treatment to current gold standard periodontal treatment to stop the reassembly of pathogenic subgingival plaque biofilm communities and subsequent failure of treatment. There are a number of barriers to the implementation of bacteriophage therapy to a chronic disease caused by a dysbiosis of endogenous bacterial communities. To realize the potential for bacteriophage therapy there must initially be a greater research effort focussed on discovery, identification, characterization and isolation of bacteriophage that target key oral bacteria essential for pathogenic dysbiotic community development. This will require the determination of the specificity of isolated and purified bacteriophage coupled with a deeper understanding of which oral bacteria are crucial for the development of the dysbiotic subgingival bacterial communities that drive disease. It is highly likely that multiple bacteriophages will need to be formulated into cocktails that may be dependent on the bacterial species present in individuals. This is likely to require the development of bacteriophage biobanks and potentially incorporation of personalized medicine approaches. In addition, effort will need to be expended to develop more applicable models of this polymicrobial chronic disease.

## Abbreviations

CAL: clinical attachment loss
CRISPR: clustered regularly interspaced short palindromic repeats
dsDNA: double stranded DNA
IMG/VR: Integrated Microbial Genome/Virus database
ICTV: International Committee on Taxonomy of Viruses
UViGs: uncultivated viral genomes

## Data sharing

Data sharing is not applicable to this article as no new data were created or analyzed in this study.
